# Genome sequence of an *Acinetobacter pittii* strain isolated from a monk parakeet with pneumonia

**DOI:** 10.1128/mra.01154-24

**Published:** 2025-07-21

**Authors:** Ivana Blancas-Nava, Rigoberto Hernández-Castro, Miguel Angel Cevallos

**Affiliations:** 1 Centro de Ciencias Genómicas, Programa de Genómica Evolutiva, Universidad Nacional Autónoma de México, Cuernavaca, Morelos, Mexico; 2 Departmento de Ecología de Patógenos, Hospital General Dr. Manuel Gea González 37762, Ciudad de México, Mexico; Wellesley College, Wellesley, Massachusetts, USA

**Keywords:** monk parakeet, *Myiopsitta monachus*, pneumonia

## Abstract

*Acinetobacter pittii* 624-B was obtained from a parakeet with pneumonia. It is resistant to cefoxitin, cephalothin, cephalexin, ampicillin, clindamycin, cefuroxime, and cefovencin. The genome has a blaADC-13 gene, an ANT(3″)-IIa gene, and a blaOXA-213-like gene. It also contains the genes for acinetobactin synthesis and for type II and VI secretion systems.

## ANNOUNCEMENT

*Acinetobacter pittii* is a microorganism that has been isolated from diverse environmental sources (1–4), including a wide range of animals. It is often found in these hosts as a disease agent (5–10). However, only one strain has been isolated from birds (11).

Strain 624-B was isolated from a monk parakeet (*Myiopsitta monachus*) with pneumonia symptoms kept in captivity in Tulancingo, Hidalgo (Mexico). The sample was obtained by inserting sterile cotton swabs into the nostrils, stroking the mucosa smoothly, and removing the sample without encountering the nasal boundaries’ skin. The swab was applied directly onto the agar medium.

The sample was cultured in MacConkey agar medium without antibiotics, and plates were incubated for 24–48 h at 37°C. The strain was purified three consecutive times on MacConkey agar plates from single colonies. The gDNA was purified from an overnight culture grown at 37°C and 250 rpm in 5 mL of Luria-Bertani liquid medium with the Genomic DNA purification kit (Thermo-Fisher), following the manufacturer’s instructions. After quantifying the DNA with a Qubit four fluorometer, two sequencing platforms were used to obtain the genome sequence of the strain: first, an Oxford Nanopore long-read library was constructed and sequenced at Plasmidsaurus Inc. The library was constructed with the Rapid Barcoding Kit 96 V14 (SQK-RBK 114.96; Oxford Nanopore Technology). The reads were obtained using a PromethION P24 with a R10.4.1. flow cell device (Nanopore Technology). The basecalling algorithm was ont-dorado-for-promethion v7.4.12 on super-accurate mode. The read quality control and adapter trimming was performed with MinKnow 23.07.1. Second, a short read library was constructed and sequenced (2 × 150 bp) using the NextSeq2000 platform at Plasmidsaurus Inc. Adapter removal and quality trimming of the Illumina reads were made using Trim Galore v0.6.4 (https://github.com/FelixKrueger/TrimGalore). The genome assembly was obtained and circularized with Unicycler v0.5.0 (12). The assembly resulted in one circular chromosome. Assembly statistics was calculated with Quast v5.0.2 (13). The completeness and contamination degree of the assembly were evaluated using CheckM 1.1.3 (14). The genome was annotated using the NCBI Prokaryotic Genome Annotation Pipeline 6.6 (15). (Table 1) The taxonomic identity of the strain was confirmed through ANI against the *A. pittii* reference strain PHEA-2 (GCF_000191145.1)(16).

**TABLE 1:** Summary of the whole-genome sequencing of *Acinetobacter pittii* 624-B

No. of reads (Illumina)	8,054,082	tRNAs	73
No. of reads ONT	341,763	ncRNA	4
ONT N50	2754	Genes	3825
No. contigs	1	BioProject	PRJNA1169018
Genome coverage	669X	Biosample	SAMN44064241

To identify the antibiotic-resistance genes encoded by this genome, we used CARD 3.3.0 (17). 624-B encodes a *bla*ADC-13 beta-lactamase, a *bla*OXA-213-like gene whose product usually hydrolyzes carbapenems weakly, and an ANT(3″)-IIa gene involved in aminoglycoside resistance. Additionally, the strain has five efflux pumps: AbeS, AdeF, AmvA, AbaQ, and AbaF that, when overexpressed, could lead to a reduction of susceptibility to several antibiotics (18). This strain has the genes involved in the synthesis of acinetobactin, a molecule required for iron uptake (19).

## Data Availability

The genome sequence has been deposited in DDBJ/EMBL/GenBank under the accession number CP185815.1. Illumina and ONT reads have been deposited in SRA with SRX26326469 and SRX26326470 accession numbers, respectively.
